# CXC Chemokine Ligand 12 Protects Pancreatic β-Cells from Necrosis through Akt Kinase-Mediated Modulation of Poly(ADP-ribose) Polymerase-1 Activity

**DOI:** 10.1371/journal.pone.0101172

**Published:** 2014-07-02

**Authors:** Nevena Grdović, Svetlana Dinić, Mirjana Mihailović, Aleksandra Uskoković, Jelena Arambašić Jovanović, Goran Poznanović, Ludwig Wagner, Melita Vidaković

**Affiliations:** 1 Department of Molecular Biology, Institute for Biological Research, University of Belgrade, Belgrade, Serbia; 2 Department of Medicine III, Division of Nephrology and Dialysis, Medical University of Vienna, Vienna, Austria; Tel-Aviv University, Israel

## Abstract

The diabetes prevention paradigm envisages the application of strategies that support the maintenance of appropriate β-cell numbers. Herein we show that overexpression of CXC chemokine ligand12 (CXCL12) considerably improves the viability of isolated rat Langerhans islet cells and Rin-5F pancreatic β-cells after hydrogen peroxide treatment. In rat islets and wt cells hydrogen peroxide treatment induced necrotic cell death that was mediated by the rapid and extensive activation of poly(ADP-ribose) polymerase-1 (PARP-1). In contrast, CXCL12-overexpressing cells were protected from necrotic cell death as a result of significantly reduced PARP-1 activity. CXCL12 downstream signalling through Akt kinase was responsible for the reduction of PARP-1 activity which switched cell death from necrosis to apoptosis, providing increased protection to cells from oxidative stress. Our results offer a novel aspect of the CXCL12-mediated improvement of β-cell viability which is based on its antinecrotic action through modulation of PARP-1 activity.

## Introduction

Diabetes is a chronic metabolic disorder characterized by hyperglycemia which results from insufficient insulin level or unresponsiveness of target cells to insulin action. While the major forms, type 1 (T1D) and type 2 (T2D) diabetes, have different aetiologies, pancreatic β-cell dysfunction and death are at the core of diabetic pathophysiology. Current strategies in diabetes management are directed at lowering blood glucose levels and treating the pathological consequences of diabetes rather than its causes. Since a common feature of diabetes is a reduction in β-cell mass, the promotion of β-cell growth and survival by therapeutic treatments is considered as a novel approach for diabetes management.

Advances in β-cell research have recently illuminated the important role of CXC chemokine ligand 12 (CXCL12) in preserving β-cell viability and regeneration. CXCL12 is a chemokine constitutively expressed in a wide range of tissues [1]. CXCL12 mediates its function through the CXCR4 [2] and CXCR7 [3], a specific G protein-coupled receptors. The CXCL12/CXCR4 axis has an important and conserved role in determining proper cell localization throughout the body and comprises the only chemokine/chemokine receptor pair that results in late embryonic lethality in mouse knockouts [4]. CXCL12/CXCR4 axis is also involved in many aspects of cell survival and tissue repair and regeneration [5]–[9]. The latter role is with potential interest in the management of diabetes in which the irreversible loss of β-cell mass is an important feature. Thus, treatment with CXCL12 protects INS-1 cells against injury induced by serum withdrawal, thapsigargin, cytokines and glucotoxicity [10]. RIP-SDF-1 transgenic mice expressing CXCL12 under the control of the insulin promoter, are to some extent protected against streptozotocin-induced diabetes, suggesting that CXCL12 agonists could provide beneficial effects in the treatment of diabetes [11]. It has been shown that CXCL12 protects and prolongs the life span of β-cells by inhibiting the apoptotic process throughout Akt and ERK1/2 activation [12].

While it is generally assumed that in both forms of diabetes β-cells primarily die by apoptosis [13], there is a growing evidence that apoptosis is not the only mechanism of β-cell death. Several *in vitro* studies revealed that β-cell necrosis is the primary mechanism by which IL-1β or combination of cytokines induces β-cell death [14], [15]. *In vivo* studies with BB rats and *Psammomys obesus* rats, model systems of T1D and T2D respectively, showed that the majority of dead islet cells exhibit a typical necrotic morphology, suggesting that necrosis is an important type of cell death during disease development [16], [17]. During the past decade, the perception of necrosis as accidental cell death has been definitively abandoned as it has been shown that necrosis, similar to apoptosis, can be a highly regulated process with important pathophysiological and therapeutic implications [18]. One of the most studied pathways in programmed necrosis is mediated via poly(ADP-ribose) polymerase-1/Diphtheria toxin-like ADPribosyltransferases (PARP-1/ARTD1) [19]. In response to severe DNA damage, prompt PARP-1 activation results in extensive poly(ADP-ribosyl)ation of target proteins. As PARP-1 uses NAD^+^ as a substrate for this reaction, hyperproduction of poly(ADP-ribose) polymers (PAR) leads to a severe depletion of cellular NAD^+^ and ATP, with the ensuing energy failure resulting in necrotic cell death [20]. The involvement of PARP-1 in β-cell death is confirmed by the observation that pharmacological inhibition or genetic deletion of PARP-1 protects animals against the development of chemically-induced diabetes and protects NOD mice from spontaneous diabetes development [21]–[23].

Bearing in mind previously established antiapoptotic effect of CXCL12, our main goal was to explore whether CXCL12 also protects pancreatic β-cells from hydrogen peroxide-induced necrotic cell death and to examine the potential mechanisms responsible for improved β-cell survival. We found that CXCL12 increases β-cell survival and redirects hydrogen peroxide-induced cell death from the necrotic to the apoptotic pathway. Lowering of PARP-1 activity is mediated by Akt kinase and is crucial for prevention of necrosis.

## Materials and Methods

### Cell culture treatment

The Rin-5F, rat pancreatic insulinoma cell line (wt) (ATCC-CRL-2058) and generated Rin-5F cells with a stable integrated human gene for CXCL12 (#1) were cultivated in RPMI medium supplemented with 10% FBS and penicillin/streptomycin (cell culture reagents obtained from PAA Laboratories GmbH, Pasching, Austria). The cell medium was exchanged every 72 h. Rin-5F wt and #1 cells were treated for 1 h (unless otherwise indicated) with 75 µM and 150 µM hydrogen peroxide, respectively. These concentrations were established to correspond to IC_50_ for each cell line. In some experiments, wt or #1 cells were pretreated with 0.5 mM 3-aminobenzamidine (3AB; Sigma, Saint Louis, MO, USA) for 30 min, 80 ng/ml of recombinant CXCL12alpha (Sigma) for 30 min, or with 10 µM AMD3100 (Sigma) for 7 days. To inhibit Akt and ERK1/2 kinases, in some experiments cells were treated with 20 µM Akt inhibitor VIII (Calbiochem, San Diego, CA, USA) or with 20 µM MEK1/2 Inhibitor UO126 (Cell Signaling, Boston, MA, USA) for 1 h, respectively.

### Isolation of Lahgerhans islets from rats

Male albino 2.5-month old (weight: 200–250 g) Wistar rats were used. The rats were kept under controlled environmental conditions of temperature (22±2°C), a 12 h light/dark cycle. Standard food pellets and tap water were provided *ad libitum*. The islet isolation technique is a slightly modified method described by [24]. Briefly, islet isolation was performed in Hank's balanced salt solution (HBSS; 115 mM NaCl, 10 mM NaHCO_3_, 5 mM KCl, 1.2 mM NaH_2_PO_4_, 25 mM HEPES, 1.1 mM MgCl_2_) containing 1% (wt/vol.) bovine serum albumin (BSA, fraction V) (Sigma) and 5 mM glucose. The pancreas was cannulated by infusion a 10 mL solution of 1.0 mg/mL collagenase in supplemented HBSS into the common bile duct. Subsequently, the distended pancreas was excised, transferred to flask tube and incubated at 37°C with 1.0 mg/mL of collagenase in supplemented HBSS for 20 min. The digestion was stopped by adding cold supplemented HBSS solution. The digest was allowed to sediment and after decanting supernatant the digest was subjected to two successive washing steps with supplemented HBSS and the resulting pellet was resuspended in the same solution. Samples of resuspended pellet were transferred to a blackened Petri dish and using a dissection microscope and an external light source, islets were handpicked (handpicking was repeated three times). Islets were collected and washed in PBS before being dispersed into single cells by mechanical shaking at 37°C for 3 min in 0.05% trypsin, 0.7 mM EDTA. The enzymatic reaction was stopped by adding RPMI 1640 culture media and after centrifugation at 400×g for 10 min, cells were washed in PBS and placed in the appropriate medium depending on the experimental protocol.

### Ethics Statement

The protocol has been approved by the Committee for Ethical Animal Care and Use of the Institute for Biological Research, Belgrade, which is in accordance with the EEC Directive (86/609/EEC) for the protection of animals used for experimental and other scientific purposes.

### MTT cell viability test

Rin-5F wt and #1 cell viability was estimated by the 3-(4,5-dimethylthiazol-2-yl)-2,5-diphenyl tetrazolium bromide (MTT; Sigma, Saint Louis, MO, USA) viability assay. Rin-5F cells were grown in 96 well plates and treated with hydrogen peroxide with or without different pretreatments as will be indicated. After removing the medium, 200 µl of MTT at a concentration of 0.5 mg/ml in RPMI was added to each well. After incubation for 2 h in the dark, the insoluble purple formazan products formed in living cells were dissolved in dimethyl sulfoxide. Absorbance was measured at 570 nm. Cell viability was expressed in percentages after comparison with control cells that were assumed to be 100% viable.

### Islet cell viability assay

Viability of islet cells was determined by simultaneous staining live and dead cells using a two color fluorescence staining: acridine orange (AO) and propidium iodide (PI). Live cells stained green, while dead cells stained red. Working solution was prepared (0.6 µl AO from 5 mg/ml and 10 µl PI from 1 mg/ml stock solutions) in 1 ml PBS. Dye mix (25 µl) was added to cell suspension and transferred to a microscope slide. Cells were visualized using Leica DMLB fluorescence microscope.

### LDH (lactate dehydrogenase) release assay

Cell culture media (without phenol red) obtained after treatment of wt and #1 cells or islet cells with increasing concentrations of hydrogen peroxide were collected, and 100 µl of the supernatant were mixed with the same volume of staining solution containing 54 mM calcium L-lactate hydrate (Sigma, Saint Louis, MO, USA), 0.66 mM iodonitrotetrazolium chloride (Sigma), 0.28 mM phenazine methosulfate (Acros Organics, Geel, Belgium) and 1.3 mM NAD (Sigma) in 0.2 M Tris-HCl, pH 7.4. The stain was developed during 15 min incubation in the dark, and the absorbance was recorded at 492 nm. The percentage of LDH release was obtained from the formula: *LDH release* (%) * = * [(*S*-*C*)/(*T*-*C*)]x100%, where S is the LDH released in cell samples, C is the level of enzyme released in the control sample, and T is the total amount of enzyme obtained after cell lysis with 3% (v/v) Triton X-100.

### Immunocytochemistry

Wt and #1 Rin-5F cells grown on glass coverslips were fixed in 4% (w/v) paraformaldehyde, permeabilized with 0.5% (v/v) Triton X-100 in PBS and blocked with 3% (w/v) bovine serum albumin (BSA) in PBS for 1 h. The slides were incubated with anti-PAR mouse monoclonal antibody (H10; Alexis Biochemicals, San Diego, CA, USA) (dilution 1∶100) overnight at 4°C in PBS-Tween 0.2% (v/v). After washing the cells in PBS-Tween 0.2%, the slides were incubated in the dark with the fluorescent secondary antibody (dilution: 1∶100) for 2 h at room temperature, washed with PBS-Tween 0.2%, and mounted on glass slides with Mowiol (Calbiochem, San Diego, CA, USA). 4,6-Diamidino-2-phenylindole (DAPI; Roche Diagnostics, Mannheim, Germany) (0.4 µg/ml) was added to visualize DNA. Slides were mounted and analyzed by fluorescence microscopy (Axio Observer Z1, Zeiss) using appropriate filters.

### Immunoblot analysis

Cell lysates were prepared with the ProteoJET Mammalian Cell Lysis Reagent (Fermentas, Burlington, Canada). Twenty µg of the cell lysate isolated from Rin-5F or islet cells or 20 µg proteins precipitated from the cell culture medium after cell treatment were separated by 12% SDS-polyacrylamide gel electrophoresis (SDS-PAGE) and electroblotted onto a polyvinylidene difluoride (PVDF) membrane. Immunoblot analysis was performed using anti-PARP-1 (H-250; Santa Cruz Biotechnology, Santa Cruz, CA, USA), anti-PAR (H10; Alexis Biochemicals, San Diego, CA, USA), anti-Akt 1/2/3 (H-136; Santa Cruz Biotechnology), anti-pAkt 1/2/3 (Ser-473-R; Santa Cruz Biotechnology), anti-ERK 1/2 (K-23; Santa Cruz Biotechnology), anti-pERK (E-4; Santa Cruz Biotechnology) antibodies. Blots were probed with horseradish peroxidase-conjugated secondary antibodies. Staining was performed by the chemiluminescent technique according to the manufacturer's instructions (Amersham Pharmacia Biotech, Amersham, UK). If different set of samples were run on different gels, immunoblot analysis was performed in the same time, under the same conditions. All membranes were exposed under the same film and thus exposure and development did not influence the obtained results.

### Immunoprecipitation

Immunoprecipitation was carried out with cell lysates (200 µg) obtained after lysis in 50 mM Tris 7.4, 150 mM NaCl, 2 mM EDTA, 1% (v/v) Triton X-100, 1 mM PMSF, 1 µg/mL aprotinin, and 1 µg/mL leupeptin (lysis buffer). Cell lysates were incubated for 2 h on ice with 0.5 µg of anti-pAkt 1/2/3 (Ser-473-R; Santa Cruz Biotechnology, Santa Cruz, CA, USA), anti-pERK (E-4; Santa Cruz Biotechnology) antibodies or equal amount of control IgG (sc-2027; Santa Cruz Biotechnology). Protein A/G-coupled agarose beads (SC-2003; Santa Cruz Biotechnology) were added for 2 h at 4°C under constant agitation. The beads were pelleted and washed five times with lysis buffer. The immunoprecipitated proteins were resuspended in the sample buffer, separated by SDS-PAGE and analyzed by immunoblot analysis.

### PARP activity assay

PARP activity was measured in nuclear lysates of wt and #1 Rin-5F cells using a commercial colorimetric PARP Assay Kit (Trevigen, Gaithersburg, MD, USA). Nuclear extracts were prepared with ProteoJET Cytoplasmic and Nuclear Protein Extraction Kit (Fermentas, Burlington, Canada) according to the manufacturer's instructions, except for the final lysis of nuclei that was performed in 0.5% Triton X-100/1xPARP buffer (from the Trevigen Kit). The PARP activity assay was performed according to the manufacturer's instructions and expressed as units of PARP-1 activity.

### Protein dephosphorylation with lambda phosphatase

Dephosphorylation was performed with 100 µg of Rin-5F nuclear lysates that were incubated for 20 min at 30°C with 400 units of lambda protein phosphatase (Sigma, Saint Louis, MO, USA) in reaction buffer supplemented with 1× MnCl_2_, all provided by the manufacturer. After the incubation, the dephosphorylated nuclear lysates were immediately used for measuring PARP activity.

### RNA isolation and quantitative real-time PCR (RT-qPCR)

Total RNA from islet cells or Rin-5F wt and #1 cells was extracted using the GeneJET RNA Purification Kit (Thermo Fisher Scientific, Waltham, MA, USA). For cDNA synthesis, 1 µg of the total RNA was treated with DNAse I and reverse-transcribed with RevertAid First Strand cDNA Synthesis Kit (Fermentas, Burlington, Canada) using oligo(dT) primers. For RT-qPCR the Maxima SYBR Green/ROX qPCR Master Mix (Fermentas) was used. mRNA levels were quantitatively determined with an ABI Prism 7000 Sequence Detection system (Applied Biosystems, Carlsbad, CA, USA). The fragments were amplified using the following primer sets: sense 5′-GATTCTTTGAGAGCCATGTC-3′ and antisense 5′-GTCTGTTGTTGCTTTTCAGC-3′ for the rat CXCL12 gene; sense 5′-ATGAACGCCAAGGTCGTGGT-3′ and antisense 5′-GGGCACAGTTTGGAGTGTTG-3′ for the human CXCL12 gene; sense 5′-CTGACTGGTACTTTGGGAAA-3′ and antisense 5′-GGAACACCACCATCCACAGG-3′ for the rat CXCR4 gene; sense 5′-ATGTGCTGCTGTATACCCTC-3′ and antisense 5′-GTGATGACGACCCACAGATC-3′ for the rat CXCR7 gene; sense 5′-CTGGTGGACATTGTGAAAGG-3′ and antisense 5′-TCTGCCTTCTGCTCAGTTTC-3′ for the rat PARP-1 gene; sense 5′-AGGCAGCGTCACCTCGTTTG-3′ and antisense 5′-GGTTCAAATACGGCGTGCTC-3′ for the rat PARG gene; sense 5′-ATGGCCCTGTGGATGCGCTT-3′ and antisense 5′-ACAATGCCACGCTTCTGCCG-3′ for rat insulin gene; sense 5′-AGATTACTGCCCTGGCTCCT-3′ and antisense 5′-ACATCTGCTGGAAGGTGGAC-3′ for the rat β-actin gene. The programme for qRT-PCR was comprised of an initial denaturation step at 95°C for 10 min and a subsequent two-step PCR programme at 95°C for 15 s, and 60°C for 60 s for 40 cycles. Negative controls without the template were used in all RT-qPCR reactions. The expression levels of the target genes were related to the averaged expression level of rat β-actin as the housekeeping gene.

### Statistical analysis

Three different samples were used and all the assays were carried out in triplicate in every experiment. Mean and standard error values (S.E.M.) were determined for all studied parameters. Results were statistically analyzed by analysis of variance (ANOVA). Duncan's multiple range test (DMRT) was performed to determine the significant differences among the groups.

## Results

### Characterization of CXCL12-overexpressing Rin-5F cells

**Figure 1:**
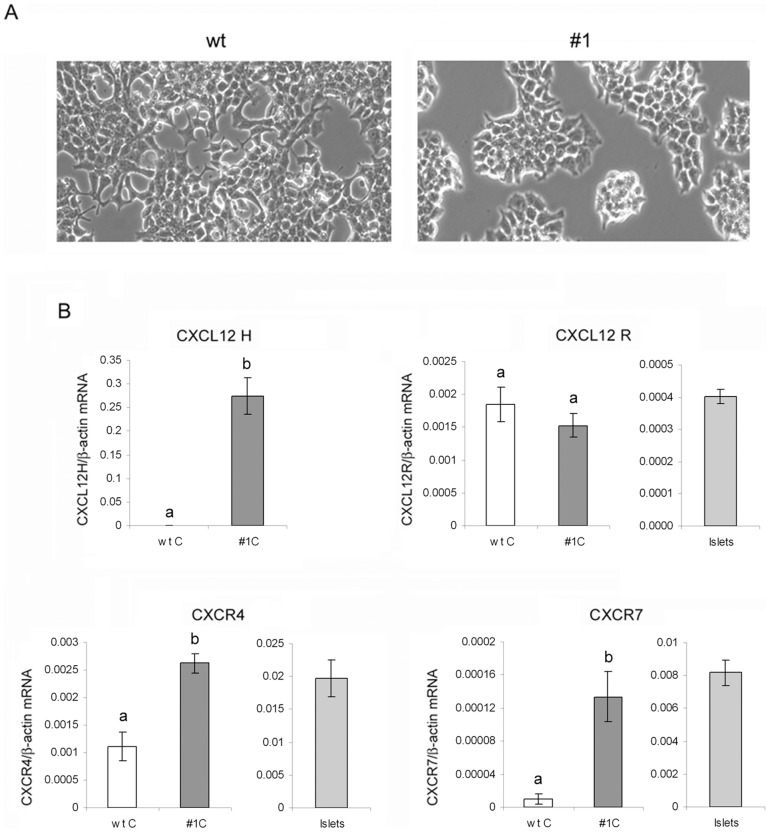
Characterization of Rin-5F cells with a stable-transfected human CXCL12 gene. (A) Wild-type Rin-5F cells (wt) and CXCL12-overexpressing Rin-5F cells (#1) observed with phase-contrast microscopy. (B) Messenger RNA levels of human and rat CXCL12, rat CXCR4 and CXCR7 receptors from Rin-5F wt and #1 cells and from isolated rat islets. The mRNA levels were determined by RT-qPCR and presented in the graphs depicting the changes in mRNA levels relative to β-actin. The results are expressed as means ± S.E.M. Means not sharing a common letter are significantly different between groups (p<0.05).

In order to explore the beneficial effects and potential mechanisms involved in CXCL12-induced β-cell survival, human CXCL12 stable-transfected rat insulinoma Rin-5F cells (referred to as #1) were used. Overexpression of CXCL12 in Rin-5F cells induced changes in morphology and growth pattern in comparison to wt Rin-5F cells. The wt cells were polygonal, exhibiting a two-dimensional, monolayer-like growth pattern, whereas CXCL12-transfected #1 cells were more spherical in shape, mainly displaying a three-dimensional, cluster-like growth pattern (Figure 1A) [25]. The levels of expression of human CXCL12, rat CXCL12, and CXCR4 and CXCR7 receptors in both cell lines, as well as in rat islets were determined by RT-qPCR (Figure 1B). In wt Rin-5F cells, endogenous CXCL12 expression was comparable with that in stable transfectants, whereas human CXCL12 was absent. In the #1 cells the expression of human CXCL12 exceeded endogenous rat CXCL12 expression 170-fold. CXCR4 expression was 2.5-fold higher in #1 than in wt cells, pointing to the likelihood of autocrine and paracrine CXCL12 signalling in stable transfectants. The expression level of the other CXCL12 receptor CXCR7 was 14-fold higher in #1 cells compared to wt cells. In islet cells expression of rat CXCL12 was quite low, while the expression levels of both receptors were dramatically higher compared to both Rin-5F cell lines (Fig. 1B).

### Susceptibility of pancreatic β-cells to hydrogen peroxide treatment

**Figure 2:**
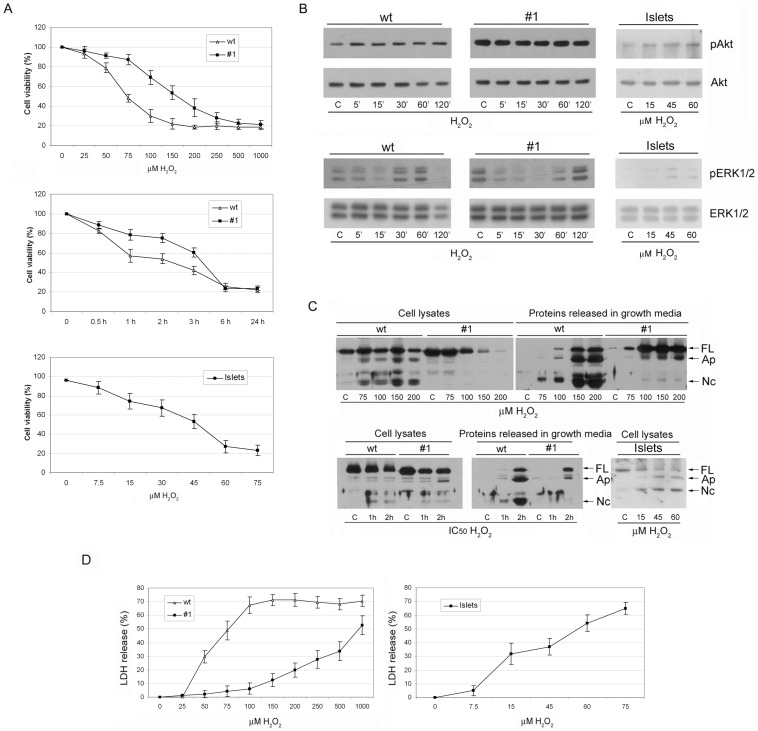
Increased expression of CXCL12 improves cell viability and switches the type of cell death. (A) Viability assay of islet cells, wt and #1 Rin-5F cells after treatment for 3 h with increasing concentrations of hydrogen peroxide or in different time points with IC_50_ of hydrogen peroxide for each cell line. (B) The effect of hydrogen peroxide treatment on the protein levels of Akt kinase and phosphorylated Akt kinase (pAkt) (top panel) and ERK1/2 kinase and phosphorylated ERK1/2 kinase (pERK1/2) (lower panel) determined by immunoblot analysis in islet cells, wt and #1 cells. (C) PARP-1 degradation pattern after treatment of islet cells, wt and #1 cells with the increasing concentrations of hydrogen peroxide or in different time points after treatment with IC_50_ of hydrogen peroxide. Immunoblot analysis with anti-PARP-1 antibody was performed with cell lysates and proteins released in cell-growth media. FL – Full length PARP-1; Ap – Apoptotic fragment of PARP-1; Nc – Necrotic fragment of PARP-1. (D) The effect of increasing concentrations of hydrogen peroxide on cell membrane integrity estimated by LDH release in islet cells, wt and #1 cells. The results are expressed as means ± S.E.M.

Susceptibility to ROS-induced cell death and the effect of CXCL12 on β-cell survival were assessed after the treatment with hydrogen peroxide. Cell viability gradually decreased with increasing concentrations of hydrogen peroxide in both cell lines and islet cells (Fig. 2A). As expected, islet cells were the most sensitive to hydrogen peroxide treatment with IC_50_ of 45 µM. However, Rin-5F #1 cells exhibited considerable resistance to hydrogen peroxide treatment in comparison with wt cells, as the IC_50_ for #1 cells (150 µM) was twice that for wt cells (75 µM). The change in cell viability with time after the treatment with IC_50_ of hydrogen peroxide for each cell line revealed different kinetics of cell death in wt and #1 cells. While more than 40% of wt cells died during the first hour of treatment, the loss of viability of #1 cells was more gradual, indicating that different mechanisms were involved in the deaths of wt and #1 cells.

### CXCL12 activates Akt and ERK1/2 in β-cells after oxidative stress induction

Since it has been shown that CXCL12 exerts its protective effects by activating the prosurvival Akt and ERK1/2 pathways, we analysed the signalling pathways that are activated in #1 cells that are growing in the presence of chronically elevated CXCL12 levels. We found that CXCL12-overexpressing #1 cells have markedly increased levels of phosphorylated Akt (pAkt) in comparison with wt cells, although the amount of Akt protein was unchanged (Fig. 2B). The treatment with hydrogen peroxide induced Akt activation in wt cells to some extent, while no additional phosphorylation of Akt was detected in CXCL12-overexpressing #1 cells which already possessed a high constitutive level of pAkt. Also, in comparison to wt cells, CXCL12-overexpressing #1 cells had a constitutively increased level of pERK1/2 (Fig. 2B). These results point to potential roles of both Akt and ERK1/2 kinases in the survival of pancreatic β-cells. The treatments with hydrogen peroxide resulted in the activation of ERK1/2 in both cell lines. In both cell lines, ERK1/2 phosphorylation decreased immediately after the treatment and gradually increased thereafter. In wt cells, phosphorylation of ERK1/2 reached a maximum at 60 min post treatment, whereas in #1 cells the highest level of ERK1/2 activation was observed after 120 min of treatment. The basal levels of pAkt and pERK1/2 kinases were very low in islet cells. Hydrogen peroxide treatment induced activation of ERK1/2 and in particular Akt kinase, indicating that the same prosurvival mechanisms are turned on in islet cells under the stress conditions.

### Increased expression of CXCL12 induces switch in the type of β-cell death

In order to establish whether the observed differences in kinetics of cell death in wt and #1 cells is due to the activation of different cell death pathways, we analysed the presence of PARP-1 cleavage products. The cleavage pattern of PARP-1 serves as a biochemical marker that can assist in distinguishing between apoptotic and necrotic types of death. After treatments of wt and #1 cells with either increasing concentrations or increasing durations of exposure to hydrogen peroxide, the cell lysates and proteins precipitated from the corresponding cell-growth media were analyzed for the presence of specific PARP-1 proteolytic fragments (Fig. 2C). In wt cells, the predominant cleavage product was the 50 kDa PARP-1 fragment, a typical marker of necrotic cell death, although the apoptotic 89 kDa fragment was also present. In contrast, in #1 cells, PARP-1 degradation yielded the apoptotic fragment as the only cleavage product, which can be particularly observed within the proteins released in growth media. The pattern of PARP-1 proteolysis in islet cells resembled the Rin-5F wt profile with predominant necrotic, but detectable apoptotic PARP-1 fragment. Different forms of cell death in response to hydrogen peroxide in wt and #1 cells were confirmed after examining LDH release (Fig. 2D). Treatments of wt cells with increased hydrogen peroxide concentrations were accompanied by a rapid raise in released LDH and induction of necrosis. In contrast, the treatment of #1 cells with IC_50_ of hydrogen peroxide induced only 10% of LDH release. A substantial increase in LDH release from #1 cells was observed only at much higher hydrogen peroxide concentrations. Therefore, in response to oxidative stress, wt cells died by apoptosis and necrosis, while the type of cell death in CXCL12-overexpressing #1 cells was undoubtedly apoptosis. Based on the LDH release, islet cells resembled the wt cells, as the treatment with IC_50_ of hydrogen peroxide induced nearly 40% of LDH release, confirming that necrosis plays important role in islet cell death induced by hydrogen peroxide.

### CXCL12 induces cell-signalling that modulates PARP-1 activity

**Figure 3:**
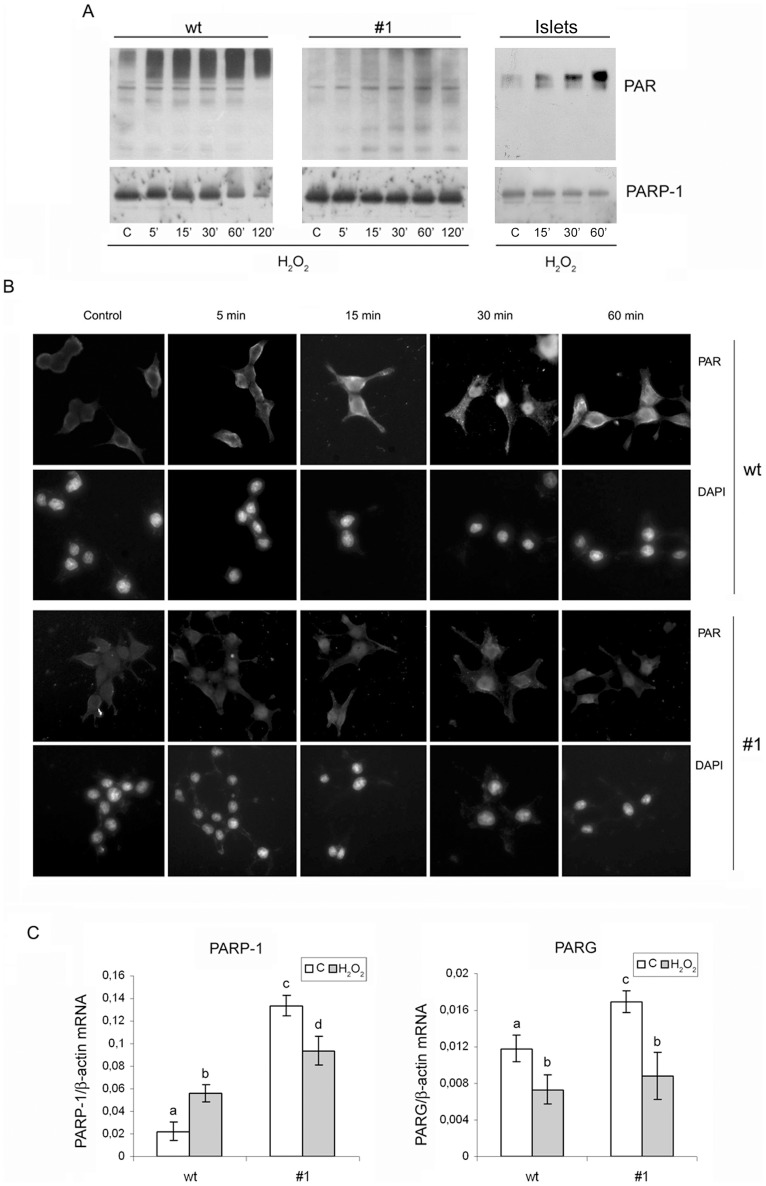
Overexpression of CXCL12 induces a diminishment of PARP-1 activity. Poly(ADP-ribosyl)ation level in islet cells, wt and #1 cells after treatment with IC_50_ hydrogen peroxide determined by immunoblot (A, top panel) and by immunofluorescent analysis in wt and #1 cells (B) with anti-PAR antibody. The PARP-1 protein level in wt and #1 cells was determined by immunoblot analysis with anti-PARP-1 antibody (A, lower panel). (C) mRNA levels of PARP-1 and PARG in wt and #1 control cells and cells after 1 h of hydrogen peroxide treatment followed by 5 h of recovery in the cell culture media. The mRNA levels were determined by RT-qPCR and presented in the graphs depicting the changes in mRNA levels relative to β-actin. The results are expressed as means ± S.E.M. Means not sharing a common letter are significantly different between groups (p<0.05).

Our next aim was to establish whether different types of cell death that were observed in the two employed pancreatic cell lines as well as islet cells, resulted from altered PARP-1 activity. Immunoblot (Fig. 3A) and immunofluorescent analyses (Fig. 3B) with antibody to poly(ADP-ribose) (PAR) polymers, which reflects PARP activity, revealed clear differences between the poly(ADP-ribosyl)ation levels in wt and #1 cells. The treatment with hydrogen peroxide induced a rapid response and extensive PARP-1 activation in wt cells. Similarly, PARP-1 was activated in response to hydrogen peroxide treatment also in islet cells. At the same time, the level of poly(ADP-ribosyl)ation was notably lower in #1 cells, with only a slight increase observed during the treatment. Immunoblot analysis (Fig. 3A) and RT-qPCR (Fig. 3C) revealed that decreased PARP-1 activity in CXCL12-overexpressing #1 cells was not the result of either lowering the PARP-1 protein level or repressing the PARP-1 gene transcription. On the contrary, the PARP-1 mRNA level was 6-fold higher in #1 than in wt cells under control conditions, and 1.7-fold higher after 1 h of treatment with IC_50_ of hydrogen peroxide. The observed lower level of poly(ADP-ribosyl)ation in #1 cells could also have been due to higher PARG activity. The level of PARG mRNA was slightly higher in #1 cells than in wt cells (1.4-fold), and could have contributed to some extent to the observed lower level of poly(ADP-ribosyl)ation in #1 cells (Fig. 3C).

**Figure 4:**
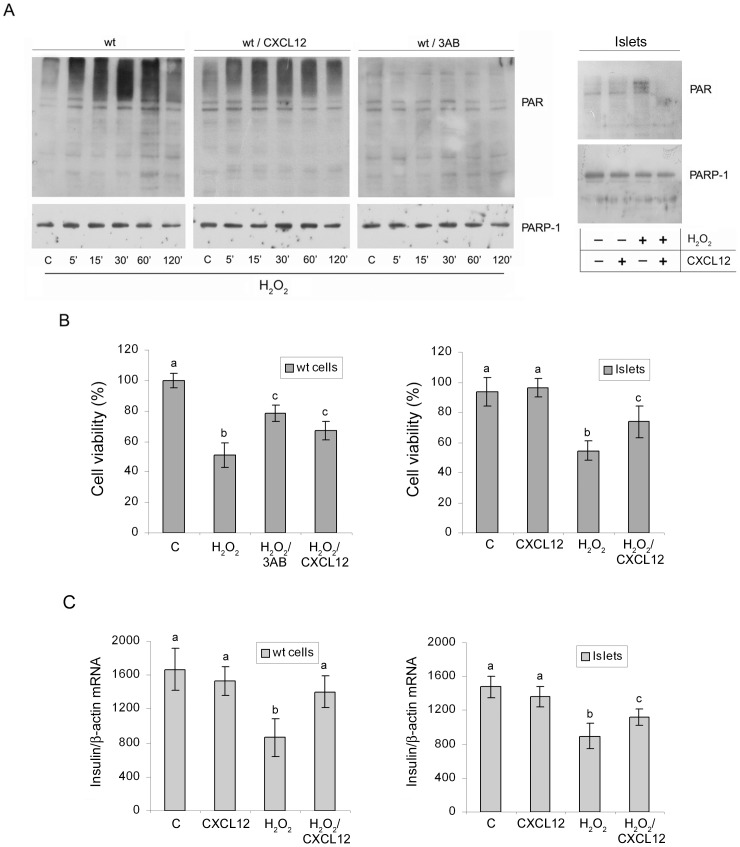
Pretreatment of β-cells with recombinant CXCL12 improves viability and induces a decrease in PARP-1 activity. (A) Poly(ADP-ribosyl)ation level of wt cells and wt cells pretreated either with recombinant CXCL12 (80 ng/ml) or with 3AB (0.5 mM) before exposure to hydrogen peroxide, determined by immunoblot analysis with anti-PAR antibody. Poly(ADP-ribosyl)ation level of islet cells before and after treatment with hydrogen peroxide and islet cells pretreated with recombinant CXCL12 (80 ng/ml) (A, top panel). The PARP-1 protein level in these samples was determined by immunoblot analysis with anti-PARP-1 antibody (A, lower panel). (B) Viability assay of control and wt cells treated with IC_50_ hydrogen peroxide and wt cells pretreated with recombinant CXCL12 or 3AB before treatment with hydrogen peroxide. Viability assay of islet cells before and after treatment with hydrogen peroxide and islet cells pretreated with recombinant CXCL12 (80 ng/ml). (C) mRNA levels of insulin wt Rin-5F and islet cells with or without pretreatment with recombinant CXCL12 before hydrogen peroxide treatment. The cells were exposed to hydrogen peroxide for 1 h, followed by 5 h of recovery in the cell culture media. The mRNA levels were determined by RT-qPCR and presented in the graphs depicting the changes in mRNA levels relative to β-actin. The results are expressed as means ± S.E.M. Means not sharing a common letter are significantly different between groups (p<0.05).

To test whether the increased level of CXCL12 in #1 is a critical factor responsible for the observed change in PARP-1 activity, wt Rin-5F and islet cells were pretreated with recombinant CXCL12 protein (80 ng/ml) before the treatment with hydrogen peroxide. Immunoblot analyses with antibody to PAR polymers revealed that the treatment with CXCL12 significantly decreased poly(ADP-ribosyl)ation in wt and islet cells (Fig. 4A). In addition, exogenously added CXCL12 improved the viability of wt cells by 17% in comparison with hydrogen peroxide-treated cells, while pretreatment with CXCL12 enhanced viability of islet cells for 20% in respect to hydrogen peroxide treated islets (Fig. 4B). Pharmacological inhibition of PARP-1 with 3AB (confirmed by immunoblot analysis with anti-PAR antibody, Fig. 4A), resulted in a 30% increase in viability of wt cells treated with hydrogen peroxide (Fig. 4B). To test whether CXCL12 pretreatment, besides viability is able to influence also the functionality of insulin-producing β-cells, insulin mRNA level was measured by RT-qPCR (Fig. 4C). We found that pretreatment with CXCL12 restored the level of insulin mRNA in wt cells after hydrogen peroxide treatment and improved insulin expression in islet cells. These results suggest that modulation of PARP-1 activity might be a novel, CXCL12-mediated mechanism for enhancing β-cell viability and functionality.

### An agonistic effect of AMD3100 on the CXCL12/CXCR4 axis

**Figure 5:**
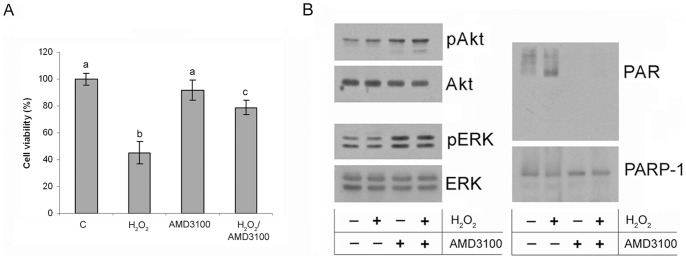
Agonistic effect of AMD3100 on CXCL12-overexpressing Rin-5F cells. (A) Viability assay of control and hydrogen peroxide-treated #1 cells grown in the absence or in the presence of AMD3100. The results are expressed as means ± S.E.M. Means not sharing a common letter are significantly different between groups (p<0.05). (B) The effects of AMD3100 treatment on the protein levels of Akt and ERK1/2 kinases and phosphorylated Akt (pAkt) and ERK1/2 kinases (pERK) (left panel), and on the poly(ADP-ribosyl)ation level and PARP-1 presence (right panel) determined by immunoblot analysis in control and hydrogen peroxide-treated #1 cells grown in the absence or in the presence of AMD3100.

AMD3100 is a CXCR4 antagonist that inhibits CXCL12 binding to the receptor and thereby blocks the downstream effects of the CXCL12/CXCR4 axis. CXCL12-overexpressing #1 cells were cultured for 7 days in a medium containing AMD3100. Shorter exposure to AMD3100 did not significantly affect cell viability and PARP-1 activity (results not shown). Unexpectedly, AMD3100 had a profound effect on #1 cell viability, improving cell survival after the hydrogen peroxide treatment by 33% in comparison with #1 cells treated only with hydrogen peroxide (Fig. 5A). This ligand-like, agonistic effect of AMD3100 was further confirmed by the increased levels of pAkt and pERK kinases observed after pretreatment of #1 cells with AMD3100 (Fig. 5B). Interestingly, AMD3100 induced complete inhibition of PARP-1 activity in both control and hydrogen peroxide treated #1 cells (Fig. 5B), suggesting once again that the activation of the CXCL12 receptors has a pronounced diminishing effect on PARP-1 activity.

### Involvement of downstream kinases in CXCL12-mediated modulation of PARP-1 activity

**Figure 6:**
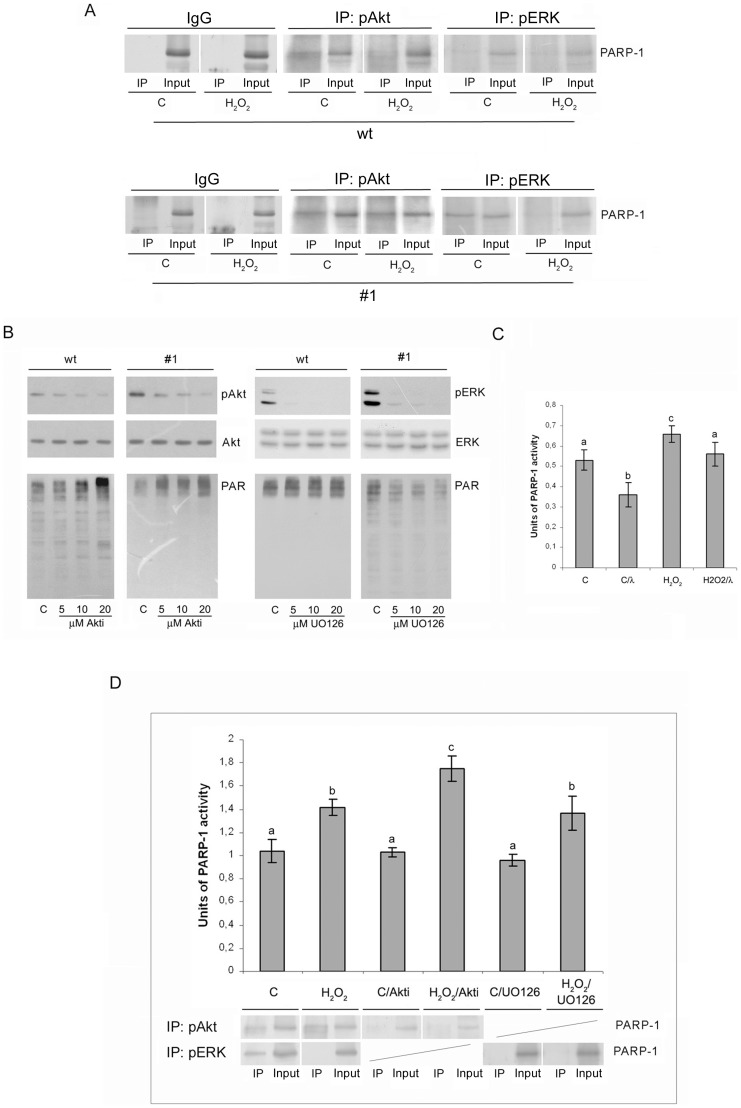
Modulation of PARP-1 activity mediated by Akt and ERK1/2 kinases. (A) Immunoprecipitation of pAkt and pERK1/2 in lysates of control and hydrogen peroxide-treated wt and #1 cells. Immunoprecipitation was performed with anti-pAkt, anti-pERK1/2 and control IgG antibodies, and the obtained immunoprecipitates were probed with anti-PARP-1 antibody. (B) The effect of Akt inhibitor VIII (left panel) and MEK1/2 inhibitor (UO126) (right panel) on the level of poly(ADP-ribosyl)ation in control wt and #1 cells using immunoblot analysis with anti-PAR antibody (lower panel). The inhibition of kinases was estimated by immunoblot analysis with anti-pAkt, anti-pERK1/2, anti-Akt and anti-ERK1/2 antibodies (top panel). (C) PARP-1 activity assay performed with nuclear lysates from control and hydrogen peroxide-treated #1 cells and lysates that were dephosphorylated by lambda-phosphatase. (D) PARP-1 activity assay performed with nuclear lysates from control and hydrogen peroxide-treated #1 cells, without pretreatment or pretreated with Akt inhibitor VIII or MEK1/2 inhibitor (UO126). Every bar graph is accompanied by a corresponding immunoprecipitate; Immunoprecipitation was performed with anti-pAkt and/or anti-pERK1/2 antibodies, and the obtained immunoprecipitates were probed with anti-PARP-1 antibody. The results are expressed as means ± S.E.M. Means not sharing a common letter are significantly different between groups (p<0.05).

Since a direct interaction of PARP-1 and kinase is a prerequisite for PARP-1 phosphorylation and change in its enzymatic activity, an immunoprecipitation assay was performed with anti-pAkt and anti-pERK1/2 antibodies in order to check for potential kinase(s)-PARP-1 protein-protein interactions. The obtained immunoprecipitates were subsequently probed with anti-PARP-1 antibody. Under physiological conditions, pAkt interacted with PARP-1 in both cell lines (Fig. 6A). In #1 cells this interaction persisted after the treatment of cells with hydrogen peroxide, while in wt cells, the oxidative insult of cells resulted in the complete disappearance of pAkt-PARP-1 interaction. No interaction between pERK1/2 and PARP-1 was detected in wt cells, neither under basal conditions nor after the hydrogen peroxide treatment. However, pERK1/2-PARP-1 interaction was detected under basal conditions in CXCL12-overexpressing #1 cells, while the hydrogen peroxide treatment resulted in a loss of pERK1/2-PARP-1 interaction (Fig. 6A). Thus, in wt cells PARP-1 activity can be modulated only in the basal state through phosphorylation by Akt. In #1 cells, both kinases can influence the activity of PARP-1 as follows: in the basal state PARP-1 can be phosphorylated with Akt and/or ERK1/2 independently, while modulation of PARP-1 activity during oxidative stress is solely the result of Akt activity.

To test if pAkt and pERK1/2 could influence the activity of PARP-1, both cell lines were pretreated with specific Akt (Akt inhibitor VIII) or MEK1/2 (UO126) inhibitors, and the corresponding cell lysates were probed with antibody to anti-PAR polymers (Fig. 6B). With increased concentration of Akt inhibitor VIII, the level of auto-poly(ADP-ribosyl)ation was augmented, suggesting that the Akt-mediated phosphorylation of PARP-1 led to a decrease in PARP-1 activity. In accordance with the absence of PARP-1-pERK1/2 interaction, the presence of MEK 1/2 inhibitor did not influence PARP-1 activity in wt cells (Fig. 6B). On the other hand, increasing concentrations of MEK1/2 inhibitor led to slight diminishment in the level of auto-poly(ADP-ribosyl)ation in #1 cells, suggesting that the phosphorylation of PARP-1 by ERK1/2 is responsible for the increase in PARP-1 enzymatic activity.

In order to estimate the overall effect of PARP-1 phosphorylation on its activity, control and hydrogen peroxide-treated CXCL12-overexpressing #1 cells were pretreated with lambda-phosphatase in order to induce protein dephosphorylation (Fig. 6C). Lambda-phosphatase dephosphorylation decreased PARP-1 activity by 30% in control, and by 15% in hydrogen peroxide-treated #1 cells, as measured by the PARP-1 activity assay, indicating that the aggregate effect of PARP-1 phosphorylation is promotion of PARP-1 enzymatic activity. Next, control and hydrogen peroxide-treated #1 cells were treated either with Akt or MEK1/2 inhibitors, after which PARP-1 activity was measured (Fig. 6D). Parallel to this, an immunoprecipitation assay was performed in order to inspect the existence of Akt-PARP-1 and ERK1/2-PARP-1 interactions, in the presence of kinase-specific inhibitors (Fig. 6D). Though treatment with Akt inhibitor promoted loss of pAkt-PARP-1 interaction it did not alter PARP-1 activity in control #1 cells. However, the pretreatment of #1 cells with Akt inhibitor before exposure to hydrogen peroxide resulted in further activation of PARP-1, in comparison with #1 cells treated only with hydrogen peroxide. Although we confirmed loss of pERK1/2-PARP-1 interaction after treatment with MEK1/2 inhibitor, (Fig. 6D, inset), the PARP-1 activity assay did not show any significant changes of PARP-1 activity as a consequence of ERK 1/2 inhibition in control #1 cells. In addition, inhibition of ERK1/2 did not influence the activity of PARP-1 after the hydrogen peroxide treatment, as expected. It can be concluded that the phosphorylation of PARP-1 mediated by Akt results in the inhibition of PARP-1 activity in #1 cells. While ERK1/2 was highly active in #1 cells, its influence on PARP-1 enzymatic activity was limited.

## Discussion

Promotion of β-cell survival is a rational therapeutic approach to prevent onset or treat diabetes. Based on protection of β-cells against different types of injury, it has been suggested that chemokine CXCL12 or its agonists could provide beneficial effects in diabetes treatment [10], [11]. The protective effects of CXCL12 have been attributed to its antiapoptotic actions. As necrosis emerges as an important mechanism of β-cell loss in diabetes, we explored the beneficial effects of CXCL12 on necrotic β-cell death. Treatment with hydrogen peroxide in Rin-5F wt and islet cells was accompanied predominantly with necrosis (Fig. 2). We found that CXCL12 present in excess amounts, either chronically as in #1 cells or transiently after treatment of wt cells with recombinant CXCL12, is responsible for the improvement of β-cell survival after exposure to hydrogen peroxide. More importantly, the same prosurvival effect was observed after pretreatment of isolated pancreatic islet cells with recombinant CXCL12.

Our results suggest that CXCL12 downstream signalling induces diminishment of PARP-1 activity, rendering the cells more resilient to the hydrogen peroxide treatment and switches cell death from necrosis, as in wt cells, to physiological cell death apoptosis detected in CXCL12-overexpressing #1 cells. Also, treatment of #1 cells with AMD3100 resulted in inhibition of PARP-1 activity and in increased viability after hydrogen peroxide treatment. Although AMD3100 is known as a specific antagonist of CXCR4 [26], [27], there are some studies pointing to partial agonistic effects of AMD3100 [28] most likely through interaction with alternative CXCL12 receptor, CXCR7 [29]. The activation of CXCR7 by its physiological ligands results in the activation of Akt [30] and ERK kinase [31], and enhances survival and proliferation of myeloma cells *in vitro* [32], [33], similar pattern of events that we observed after the AMD3100 treatment. Regardless of the cause of the AMD3100-induced receptor(s) activation, our overall finding is that stimulation of CXCL12 receptor(s) resulted in inhibition of PARP-1 activity.

Recently, several kinases were identified as new regulators of PARP-1 activity. Depending on the kinase involved, the phosphorylation of PARP-1 can result in either augmentation or inhibition of its enzymatic activity. Protein kinase C (PKC) [34] and DNA-dependent protein kinase (DNA-PK) [35] phosphorylate and inhibit PARP-1 activity, while PARP-1 phosphorylation mediated by 5′ AMP-activated protein kinase (AMPK) [36], ERK1/2 [37] and c-Jun N-terminal kinase (JNK) [38] promote the catalytic activity of PARP-1. The prosurvival effect of CXCL12 was proposed to be through the activation of Akt and ERK kinases [12]. In our experimental setup, overexpression of CXCL12 increased the level of both pAkt and pERK1/2 in #1 cells when compared to wt cells, pointing to a potential role of both signalling pathways in the increased survival rate of pancreatic β-cells. Immunoprecipitation experiments revealed that both CXCL12-downstream kinases Akt and ERK1/2 can contribute to a change in PARP-1 activity in CXCL12-overexpressing #1 cells. Interestingly, we observed a complete loss of pERK1/2-PARP-1 interaction after the treatment of #1 cells with hydrogen peroxide, while the interaction of pAkt and PARP-1 persisted after the insult. In wt cells, PARP-1 was found to interact only with Akt kinase under basal conditions. Although the results we obtained after complete protein dephosphorylation using lambda-phosphatase suggest that the sum effect of PARP-1 phosphorylation in #1 cells is promotion of its activity, experiments with the specific Akt inhibitor unambiguously showed that the result of Akt-PARP-1 interaction is inhibition of PARP-1 activity. Although there is no literature data about Akt-mediated PARP-1 phosphorylation, a similar effect on PARP-1 activation in LPS-stimulated macrophages was observed after the incubation of cells in the presence of the PI3K inhibitor LY294002 [39]. Using MEK1/2 inhibitor, we found that the ERK1/2-mediated phosphorylation of PARP-1 induces statistically insignificant increase in PARP-1 activity under both conditions.

**Figure 7:**
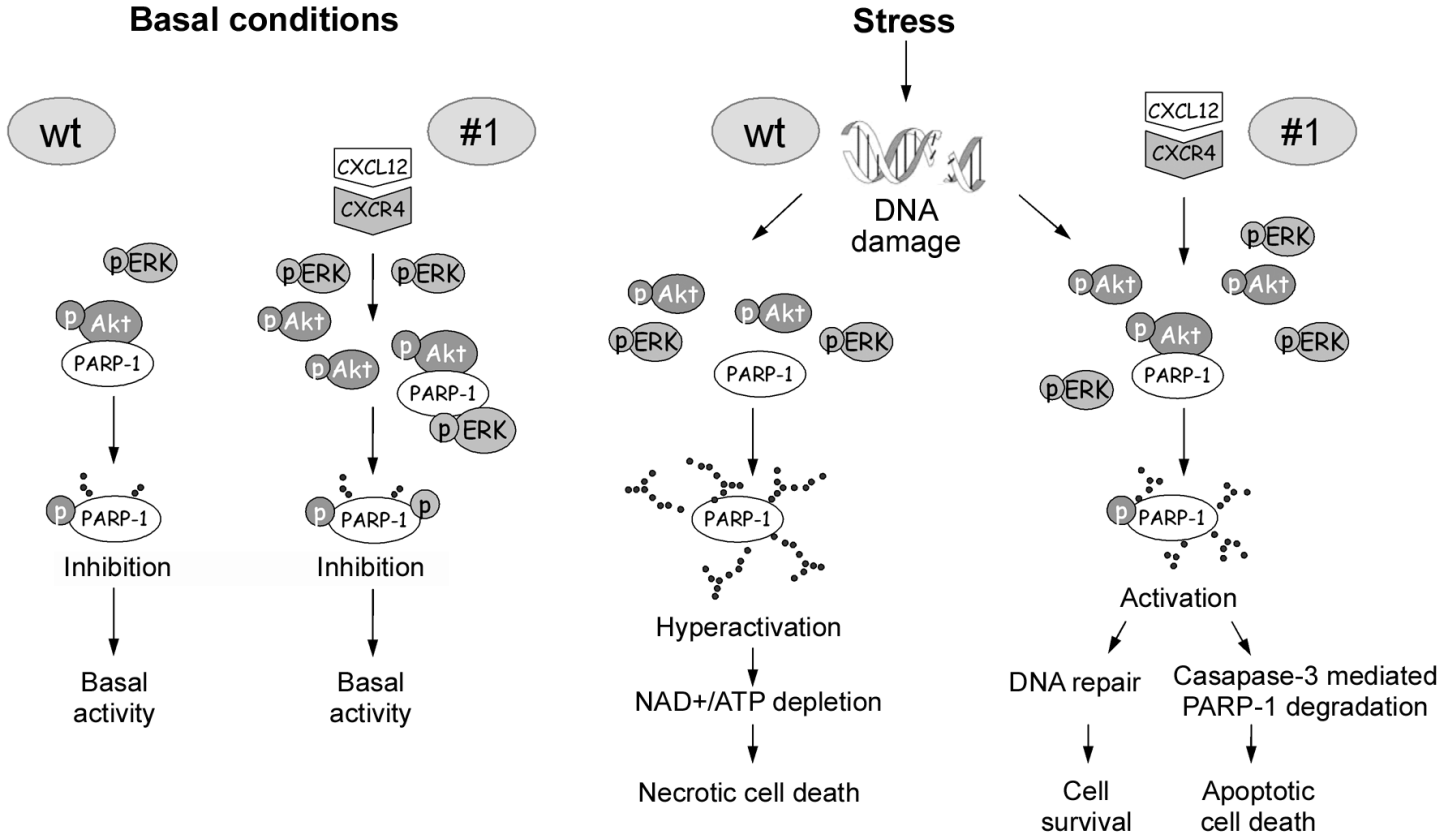
Antinecrotic effects of CXCL12 in pancreatic β-cells. During the basal conditions in wt Rin-5F cells, PARP-1 interacts with activated pAkt. This interaction allows for PARP-1 phosphorylation that results in PARP-1 partial inhibition. In the increased presence of CXCL12 (#1) activated kinases, pAkt and pERK1/2 are found to be PARP-1 interacting partners with no additional influence on its enzymatic activity. In contrast, during the stress conditions and in response to severe DNA damage in wt cells, the loss of pAkt-PARP-1 interaction lifts the suppression on PARP-1 enzymatic activity, allowing for extensive PARP-1 auto-poly(ADP-ribosyl)ation, NAD^+^ and ATP depletion and final necrotic cell death. The excess of CXCL12 in #1 cells allows for the increased phosphorylation of Akt kinase and pAkt-PARP-1 interaction. This interaction persists even after an insult has passed, maintaining a continual partial suppression of overactivated PARP-1 that results in moderately active PARP-1. In this scenario, cellular energy depletion has been prevented and switch from the necrotic to the apoptotic death has been secured by pAkt-induced prevention of PARP-1 overactivation. With the fine-tuned suppression of overactivated PARP-1 in cell under the stress, cell still operates with the active PARP-1 that is essential player in many cellular processes.

Based on these findings we proposed a working model (Fig. 7) which additionally explains CXCL12-mediated improvement of β-cell viability. According to this, the interaction of pAkt with PARP-1 and its subsequent phosphorylation has a crucial impact on maintaining low PARP-1 activity. In response to severe DNA damage, the loss of pAkt-PARP-1 interaction lifts the suppression on PARP-1 enzymatic activity, allowing for extensive PARP-1 auto-poly(ADP-ribosyl)ation, NAD^+^ and ATP depletion and results in necrotic cell death. The excess of CXCL12 present in #1 cells allows for the activation of downstream signalling kinases (Akt and ERK1/2) through paracrine and autocrine actions. The subsequent phosphorylation status of PARP-1 after the hydrogen peroxide treatment that is mediated by both kinases, contributes to the prevention of hyperactivation of PARP-1 in #1 cells. Namely, the increased phosphorylation of Akt kinase induces pAkt-PARP-1 interaction which persists even after an insult has passed, maintaining a continual partial suppression of PARP-1 activity. The prevention of PARP-1 hyperactivation in the presence of CXCL12 is also assisted by the loss of the PARP-1 stimulating interaction with pERK. The proposed modulation of PARP-1 activity by CXCL12 could be a novel mechanism that additionally explains the prosurvival effects of CXCL12. Although pharmacological inhibitors of PARP-1 improve β-cell resistance against various injury agents [21], the complete inhibition of this enzyme with so many different roles in cell metabolism could be deleterious. CXCL12-mediated reduction of PARP-1 activity, in contrast to its total inhibition, would allow the repair of damaged DNA and basic transcriptional activity of PARP-1 in cells exposed to stress. At the same time, if DNA damage is too widespread, it would allow apoptosis since PARP-1 hyperactivation, energy depletion and activation of necrotic cell death is prevented (Fig. 7).

Akt kinase is well known for its antiapoptotic prosurvival effects. According to our results, it also possesses an antinecrotic potential. It was previously reported that the activation of Akt inhibits ceramide-induced cell death with a necrotic morphology in glioma cells [40]. Also, the expression of active Akt kinase protects against ischemia-reperfusion-induced necrosis in rat liver and cardiomyocytes [41], [42], as well as against nephrotoxicant-induced necrosis in renal proximal tubule cells [43]. Although the mechanism of the Akt-mediated antinecrotic effects was not established, it was shown that Akt activation protects against necrosis by maintaining intracellular ATP levels [43], [44], implying a potential role of PARP-1 in this process. It is possible that the Akt-mediated inhibition of PARP-1 activity and thereby maintenance of ATP levels in the cell, the mechanism we propose as an antinecrotic effect in β-cells, is a common mechanism in Akt-mediated protection against non-apoptotic cell death.

Recently, Liu and co-workers postulated a feed-back loop between CXCL12 secretion from injured pancreatic β-cells and glucagon-like peptide-1 (GLP-1) production in α-cells that induces the growth and survival of β-cells [10] and trans-differentiation of α-cells into new β-cells [45]. It was shown that GLP-1, besides its antiapoptotic properties, has the potential to protect pancreatic β-cells against necrotic cell death induced by cytokines, and that these protective effects require the activation of Akt kinase [46]. In the present study, CXCL12 was shown to protect pancreatic β-cells from necrotic cell death by diminishing PARP-1 activity in an Akt-dependent manner. GLP-1 is already in use as a therapeutic agent in T2D patients because of its effect on glucose metabolism. Treatment with a combination of GLP-1 and CXCL12 would not only stimulate insulin secretion, but could also exert a direct effect on pancreatic β-cells, enhancing their mass and promoting their survival. Aside from its potential to stimulate β-cell regeneration and prevent apoptosis, capability of CXCL12 to prevent pancreatic β-cells from entering the necrotic cell death by modulating PARP-1 activity, places chemokine CXCL12 in the focal point of developing strategies for diabetes treatment.

In conclusion, in this paper we shed light on additional mechanisms besides previously established, which could further explain the increased viability of rat pancreatic islet cells as a result of CXCL12 signaling. We showed that chemokine CXCL12 improves pancreatic β-cell and islet cells survival after hydrogen peroxide treatment and if the damage is too severe, switches cell death from necrotic to apoptotic. We found that the observed prevention of necrosis results from reduced PARP-1 activity and that diminishment of PARP-1 activity is mostly mediated by its phosphorylation via activated Akt kinase. Crosstalk between PARP-1 and Akt kinase could explain fine-tuned modulation of PARP-1 activity by CXCL12 and additionally clarify the prosurvival, in particular antinecrotic effects of CXCL12 on pancreatic β-cells. We believe that the antinecrotic effect of CXCL12 is particularly important in diabetes as it prevents the generation of an additional pro-inflammatory burden resulting from β-cells proceeding along the necrotic pathway.
